# Genome-wide copy number variant discovery in dogs using the CanineHD genotyping array

**DOI:** 10.1186/1471-2164-15-210

**Published:** 2014-03-19

**Authors:** Anna-Maja Molin, Jonas Berglund, Matthew T Webster, Kerstin Lindblad-Toh

**Affiliations:** 1 Department of Animal Breeding and Genetics, Swedish University of Agricultural Sciences, Uppsala, Sweden; 2 Science for Life Laboratory, Department of Medical Biochemistry and Microbiology, Uppsala University, Uppsala, Sweden; 3 Broad Institute of Harvard and Massachusetts Institute of Technology, Cambridge, Massachusetts, USA

**Keywords:** Copy number variation, CNV, SNP genotyping array, Dog genome, Deletion, Duplication, CanineHD

## Abstract

**Background:**

Substantial contribution to phenotypic diversity is accounted for by copy number variants (CNV). In human, as well as other species, the effect of CNVs range from benign to directly disease-causing which motivates the continued investigations of CNVs. Previous canine genome-wide screenings for CNVs have been performed using high-resolution comparative genomic hybridisation arrays which have contributed with a detailed catalogue of CNVs. Here, we present the first CNV investigation in dogs based on the recently reported CanineHD 170 K genotyping array. The hitherto largest dataset in canine CNV discovery was assessed, 351 dogs from 30 different breeds, enabling identification of novel CNVs and a thorough characterisation of breed-specific CNVs.

**Results:**

A stringent procedure identified 72 CNV regions with the smallest size of 38 kb and of the 72 CNV regions, 38 overlapped 148 annotated genes. A total of 29 novel CNV regions were found containing 44 genes. Furthermore, 15 breed specific CNV regions were identified of which 14 were novel and some of them overlapped putative disease susceptibility genes. In addition, the human ortholog of 23 canine copy number variable genes identified herein has been previously suggested to be dosage-sensitive in human.

**Conclusions:**

The present study evaluated the performance of the CanineHD in detecting CNVs and extends the current catalogue of canine CNV regions with several dozens of novel CNV regions. These novel CNV regions, which harbour candidate genes that possibly contribute to phenotypic variation in dogs or to disease-susceptibility, are a rich resource for future investigations.

## Background

About a decade ago the first large catalogs of copy number variants (CNVs) in the human genome were presented [1,2]. Numerous studies later, CNVs are known to contribute to the genomic variation to a larger extent than single nucleotide polymorphisms (SNPs) in terms of number of nucleotide differences [3,4]. CNVs are defined as DNA segments of variable length, up to several megabases (Mb), that varies in copy numbers in comparison to a reference genome [5]. The different types of CNVs include deletions and duplications, while consequences of CNVs include *e.g.* altered gene dosage and regulation, changed gene structure and unmasking of recessive alleles [5]. The effect of CNVs varies from being benign or neutral, to having subtle effects on disease predisposition or directly causing disease. The contribution and importance of CNVs for phenotypic diversity and disease susceptibility has been repeatedly shown in human and several complex diseases such as psoriasis, Crohn’s disease, type 2 diabetes, coronary heart disease among others have been associated with CNVs [3,6-9]. Also for the group of patients with intellectual disability and/or multiple congenital anomalies, CNVs have been shown to be of major importance and explain about 10-20% of these patients [10].

Today, genome-wide screens for CNVs have been conducted in many different species in addition to human, including mouse, pig, cattle, goat and horse [11-22]. These screens, both in human and other species, were enabled by methods to perform high-throughput genome scans. Several methods exist to search genomes for the presence of CNVs, *e.g.* array-based methods such as array comparative genomic hybridization (aCGH) and whole-genome SNP-genotyping arrays (SNP arrays). The higher the resolution of the arrays the smaller CNVs can be detected with more accuracy and precision. The advantage with SNP arrays is that CNV detection can be performed in conjunction with genome-wide association studies (GWAS) and specific algorithms have been developed to identify CNVs using SNP arrays [23-25]. More recently the next-generation sequencing technologies have emerged to detect CNVs. The necessity for cataloguing CNVs also in animals is evident considering the numerous examples of CNVs involvement in shaping the phenotype in animals [26]. For example, the pigmentation phenotype dermal hyperpigmentation/Fibromelanosis in chicken is caused by a complex rearrangement of the *EDN3* locus that involves two duplications [27]. In dogs, the dorsal hair ridge in Rhodesian and Thai Ridgeback is caused by a duplication encompassing three genes, *FGF3*, *FGF4* and *FGF19*, and this CNV predisposes to dermoid sinus [28]. Also, the wrinkled skin in Chinese Shar-Pei is explained by a CNV, namely a duplication upstream of the gene encoding Hyaluronic acid synthase 2 [29]. Similarly as in ridgebacks, the CNV in Shar-Pei predisposes the breed to a disease, namely periodic fever syndrome [29]. One example of a CNV that has been suggested to confer an advantage in early dogs during domestication is a 8 kb duplication encompassing the amylase gene *AMY2B*[30]. This duplication was one of the identified selection signals involving genes in starch digestion and it was shown to increase amylase activity suggesting that this duplication contributed to the ability of dogs to thrive on a starch-rich diet in comparison to wolves that lack the duplication [30].

In dogs, four CNV screens have previously been performed with the aim of identifying canine CNVs and all were based on the aCGH method [31-34]. The analyses by Chen *et al*. of nine dogs from different breeds identified 60 CNV regions (CNVRs, *i.e.* region of the genome where CNVs have been identified) using an array with an average probe spacing of 4,675 bp [32]. The contemporary study by Nicholas *et al*. was directed towards segmentally duplicated regions of the genome and 137 Mb of sequence was studied at a very high resolution (200 bp probe spacing) [34]. This study identified 678 CNVRs using 17 dogs (all from different breeds) and a gray wolf [34]. Recently two studies were reported using the same aCGH that has the highest resolution of existing canine arrays up to date with 2.1 million probes (1 kb probe spacing) [31,33]. One of these studies used nine dogs and a gray wolf and identified 403 CNVRs [33] and the hitherto most comprehensive CNV screen in dogs was based on 50 dogs from 17 breeds and 3 wolves and identified 430 CNVRs [31]. In 2011, the development of a high-density canine SNP array (the Illumina CanineHD array) containing 174,943 SNPs with an average probe spacing of 13 kb was reported [35]. This array enables CNV detection simultaneously with SNP genotyping, although the CanineHD array was not evaluated with respect to CNVs in that study [35]. In the present study, the first CNV screen based on the CanineHD array is reported. Using 351 dogs from 30 different breeds, the present study includes the largest number of dogs screened for CNVs hitherto. This investigation illustrates the usefulness of the CanineHD array in CNV detection and demonstrates the size-range of CNVs that are detectable with the CanineHD array. Furthermore, this study identified novel CNVRs including some breed-specific CNVRs.

## Results and discussion

### CNV discovery and genotyping

Genome-wide CNV analysis was conducted on 359 dogs from 30 different breeds (Additional file 1: Table S1) using the Illumina 170 K CanineHD SNP array [35,36]. A total of 26 breeds included 7 or more samples, enabling a thorough identification of single breed CNVs, *i.e.* CNVs present in one breed exclusively. Importantly, 13 breeds had never been analyzed genome-wide for the presence of CNVs before and an additional five breeds had only been genotyped for previously identified CNVs in the study by Nicholas *et al*. [33]. Therefore, the present study enabled also the identification of novel CNVs.

Briefly, the initial CNV discovery was performed using two different algorithms, PennCNV and QuantiSNP [23,24,37,38]. After quality filtering, 351 dogs remained and the relaxed QuantiSNP result file (Log Bayes factor; LBF ≥ 10) contained 1748 CNVs, the stringent QuantiSNP result file (LBF ≥ 30) contained 493 CNVs and the PennCNV file contained 1455 CNVs. In the quality-filtering step, three large regions of a total size of 9.2 Mb containing 59 genes (Additional file 2: Figure S1 and Additional file 1: Table S2) were excluded due to extensive segmentation of the data creating many overlapping CNV calls. Thorough investigations of these regions were beyond the scope of this study but this observation is of importance in future CNV studies based on the CanineHD array.

The identification of CNV regions (CNVRs) was performed by using a stringent procedure. Overlapping CNVs were merged from the two programs separately, *i.e.* the stringent list of 493 CNVs identified by QuantiSNP were merged into 116 CNVRs and the 1455 CNVs found by PennCNV yielded 261 CNVRs. The breakpoints of each of these CNVRs were defined by the outer boundaries of the individual CNVs. Finally, the CNVRs from QuantiSNP that did not overlap with CNVRs from PennCNV, with at least 10 kb overlap were excluded. This identified a stringent list of CNVRs where the breakpoints of each of these final CNVRs were obtained from the QuantiSNP CNVRs. The strategy where the final list of stringent CNVRs was found by both algorithms has been recommended previously in order to reduce the false discovery rate [25].

Altogether 110 CNVRs, distributed on the 38 autosomes, were identified following this process (Additional file 1: Table S3). For these stringent CNVRs, each of the 351 individuals were genotyped using the relaxed QuantiSNP file with 1748 CNVs, hence a less strict threshold was used in CNV genotyping than in CNV discovery (LBF ≥ 10 *vs*. ≥ 30). Of these 110 CNVRs, 38 were found in only one single sample and thereby denoted singletons (Additional file 1: Table S3). The frequency of singletons in the present study was 0.11 (38/351) singletons per sample which confirm the observation by Berglund *et al*. of a low number of singletons per sample as compared to the conflicting results reported in Nicholas *et al.*[31,33]. Since singletons are more likely to be false-positives than CNVRs that are detected in at least two independent individuals, a conservative approach was selected where singletons were excluded from further analysis. However, future CNVR screens of additional individuals could reveal if some of these singletons (Additional file 1: Table S3) in fact represent rare CNVRs instead.

**Figure 1:**
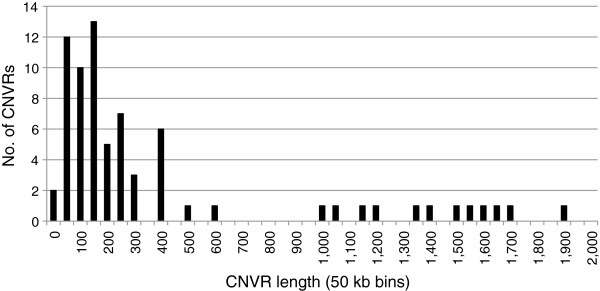
**Size distribution of the 72 identified CNVRs.** The number of CNVRs were divided into 50 kb bins so that those CNVRs with a size between 0 to <50 kb are in the first bin denoted 0, those CNVRs between 50 kb to <100 kb are in the second bin denoted 50 and so forth. On the x-axis the bins are denoted using the shortest size of the bin.

The 72 CNVRs left after removal of singletons included 31 CNVRs that were defined as pure deletions (*i.e.* only heterozygous or homozygous deletions were found among the 351 samples), 30 CNVRs were defined as pure duplications (*i.e*. only heterozygous or homozygous duplications were found among the 351 samples) and 11 as being either del/dupl meaning that both deletions or duplications were observed among the 351 samples. Regarding the 11 CNVRs defined as del/dupl, 80% of the samples with a CNV harboured a duplication. The median length of all CNVRs was 195 kb and upon subdivision in deletions and duplications, the median length of deletions was 155 kb and for duplications 282 kb (Table 1). Deletions were smaller than duplications (p-value = 0.0006, two-sample t-test), as in a previous study [31]. It has been hypothesized that the size difference is due to deletions being more deleterious to the genome than duplications and that there is selection against deletions [31]. The smallest CNV detected in this study was a 38 kb deletion, which was one of the CNVRs verified by aCGH. This deletion showed a concordant telomeric endpoint but a deviating centromeric endpoint compared to the endpoints defined in the previous study where the size of the deletion, as defined by aCGH, was 63 kb [31]. By using the CanineHD array with a resolution of 1 probe/13 kb, the distribution of identified CNVRs were as follows: 19% (14/72) were smaller than 100 kb, about 64% (46/72) of the CNVRs were in the range of 100 kb – 1 Mb and 17% (12/72) were larger than 1 Mb in size (Figure 1).

**Table 1:** Summary of identified CNVR

	Total CNVRs*		
Number of CNVs	72		
Median length	194,559		
No. of genes	148		
No. of CNVs with genes	38		
	**Del**	**Dupl**	**Del/Dupl**
Number of CNVs	31	30	11
Median length	154,574	281,565	447,710
No. of genes	23	48	77
No. of CNVs with genes	8	21	9

*after removal of the 38 singletons, the remaining CNVRs were 72.

### Comparison with published CNVRs and validation of CNVRs

The currently identified 72 CNVRs were compared to CNVRs defined in two previously published datasets to look for overlap between the dataset. As threshold for overlap, a minimum of at least 10 kb overlap between the currently identified CNVRs and a previously published CNVR was required [31,33].

Using this strategy, altogether 60% of the 72 CNVRs were shown to overlap previously identified CNVRs (Table 2). However, since the present investigation contained almost six times more samples than previous screens for CNVRs, the possibility of detecting rare CNVRs in this study was considerably higher. Considering the difference in sample size, the CNVRs in this study were subdivided into three categories; CNVRs with a frequency of >5% in the population, CNVRs with a frequency of >1% and finally the rare CNVRs that were present in <1% of the screened samples. This subdivision revealed that of the CNVRs present in >5% of the population all overlapped with previously published CNVRs and as many as 73% of the CNVRs with a frequency of >1% overlapped (Table 2). In the last category of CNVRs with a frequency of <1% in the population, only 33% overlapped known CNVRs.

**Table 2:** Comparison with previously identified CNVRs using a minimum of 10 kb overlap

	**Number of CNVRs**	10 kb overlap*
All CNVRs	72	43 (60%)
CNVRs with frequency >5%	11	11 (100%)
CNVRs with frequency >1%	48	35 (73%)
CNVRs with frequency <1%	24	8 (33%)

*Previously published datasets were [31,33].

The high proportion of CNVRs with a frequency of at least 1% in the population that overlap with previously found CNVRs suggests a high accuracy in the currently described CNV discovery procedure. In fact, an overlap of 73% is higher than the observed reproducibility of <70% for most platforms when the same sample has been analyzed in replicate experiments [39].

Given that a majority of CNVRs identified herein were supported by previously identified CNVR datasets, a subset of CNVRs was selected for validation only among the novel CNVRs/single breed CNVRs (see "CNVRs in single breeds" and "Dosage sensitive genes and novel CNVRs"). Five deletions and four duplications with a size range from 52 to 343 kb were investigated by quantitative real-time PCR (qRT-PCR) in 28 samples from three breeds (Additional file 1: Table S4). Eight of the nine CNVRs were validated yielding a validation rate of 89% (Additional file 1: Table S4). The unconfirmed CNVR, no. 45, was a deletion present in only 2/351 individuals and only one sample was available for validation. Based on visual inspection of the Log R ratio and the B allele frequency plots, the unavailable sample showed a more convincing deletion than the qRT-PCR-investigated sample (Additional file 3: Figure S2) suggesting that CNVR no. 45 may still be a true CNVR. The genotype concordance between the CanineHD array data and the qRT-PCR validation was 98%. Overall 106 sample-locus combinations were tested, where 20 of these displayed a deletion or duplication in the CanineHD CNV genotype. The results showed that two sample-locus combinations did not match (one false positive and one false negative, Additional file 1: Table S4). In summary, both the validation rate of CNVRs of 89% and the genotyping concordance rate between the two methods of 98% suggest that the described CNV discovery and genotyping procedure is of high accuracy and that the performance of the CanineHD array in detecting CNVs is high.

### CNVRs in single breeds

**Figure 2:**
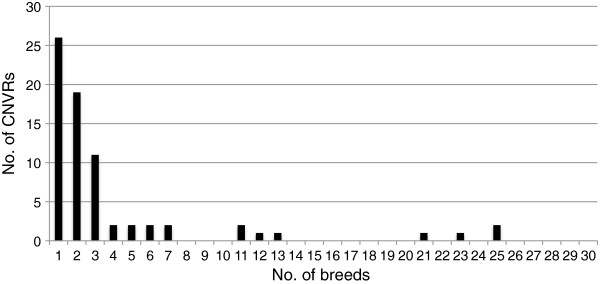
**Distribution of the number of CNVRs among breeds.** Altogether 26 CNVRs affect one breed, 19 CNVRs affect 2 breeds and the OR containing CNV (CNVR no. 52) on chromosome 14 affects as many as 25 breeds.

The strengths of this study are the high number of samples per breed and the large number of different breeds, which facilitates the identification of single breed CNVRs more accurately than in previous studies. The majority of the 72 CNVRs were present in a small number of breeds (less than four breeds) and 26 CNVRs were present in one breed exclusively; *i.e.* single breed CNVRs (Figure 2, Additional file 1: Table S5). By comparing with the two previously published CNVR datasets [31,33], some of the 26 single breed CNVRs had been previously found in one other or multiple other breeds before and where therefore removed from the list of single breed CNVRs along with a few CNVRs where previous studies lacked information about which breed was affected (Additional file 1: Table S5). Consequently, a total of 15 strictly defined single breed CNVRs remained and these were found in 12 different breeds (Table 3). Likewise, in the study by Berglund *et al*., 78 single breed CNVRs had been identified, but 10 of these were found in other breeds when comparing with the 110 presently identified CNVRs (Additional file 1: Table S3). Hence the single breed CNVRs defined by Berglund *et al*., was reduced to 68 single breed CNVRs [31]. This illustrates that further investigations are necessary to fully understand which CNVRs are found in which breed and which CNVRs are true single breed CNVRs.

**Table 3:** The stringent list of 15 single breed CNVRs

**CNVR no.**	**Chr**	**Start**	**End**	**Length**	**No. of genes**	**Type**	**Breed**	**No. of affected**	**No. of screened**	**Freq. in breed**	Genes**
78*	21	40,580,407	40,742,936	162,529	1	del	Finnish Spitz	4	12	0.33	**PDE3B**
2	1	65,361,010	65,528,809	167,799	1	del	Elkhound	2	12	0.17	TRDN
16	3	93,757,480	94,035,855	278,375	2	dupl	English Bulldog	2	12	0.17	**ANAPC13,** LCORL
23	4	82,705,831	82,993,500	287,669	0	del	Weimaraner	8	26	0.31	
37	8	28,122,926	28,265,491	142,565	0	del	Greenland Sledge	3	12	0.25	
47	13	14,209,388	14,411,209	201,821	0	del	Brittany Spaniel	5	12	0.42	
48	13	21,103,428	22,714,370	1,610,942	11	dupl	Standard Poodle	2	12	0.17	**DSCC1, TNFRSF11B, ENPP2, COLEC10, NOV, TAF2, MRPL13, DEPTOR, COL14A1, MTBP, SNTB1**
57	15	37,836,364	37,940,678	104,314	1	dupl	Shar Pei	2	11	0.18	NDUFA12
58	16	45,458,552	45,682,702	224,150	0	del	Border Collie	2	9	0.22	
68	19	13,325,414	13,514,903	189,489	0	del	Eurasian	3	11	0.27	
69	19	13,816,712	15,228,649	1,411,937	3	dupl	Shar Pei	2	11	0.18	**C4ORF33, SCLT1,** PHF17
71	19	29,002,373	30,541,395	1,539,022	1	dupl	Border Collie	3	9	0.33	CNTNAP5
82	22	21,232,747	21,306,657	73,910	0	dupl	Eurasian	3	11	0.27	
96	32	37,679,695	37,939,968	260,273	0	del	Dalmatian	2	7	0.29	
103	35	23,792,470	23,921,812	129,342	0	del	German Shepherd	3	11	0.27	

*The single breed CNVR no. 78 was previously found by Berglund *et al.* in the same breed [31].

**Genes in bold are completely within the estimated CNVR boundaries, see also Additional file 1: Table S6.

Single breed CNVRs are of particular interest if it is fixed in the breed since it could be involved in breed specific characteristics. In the study by Berglund *et al.* no fixed single breed CNVRs were identified in the two 10-sample breeds included in the study and they suggested that breed specific CNVRs are most often not involved in breed specific characteristics [31]. In the stringent list of 15 single breeds CNVRs identified herein (Table 3), the frequencies of the CNVRs were in the range of 17-42%, hence our results confirm previous suggestions [31]. The frequency of CNVRs in a breed is however not an exact measurement since it can depend on *e.g.* the discovery procedure, filtering criteria and whether the size of the CNVR is close to the detection limit of the array.

### CNVRs and the effect on genes

Examining the CNVRs for the presence of genes, the list of 24,660 genes from Ensembl CanFam2 was filtered for human orthologous genes leaving 17,239 genes [40]. Furthermore, genes located on chromosome X, unknown or mitochondria were first removed and subsequently all non-unique dog entries and all non-unique human entries leaving a total gene list of 16,305 1:1 human-dog orthologous genes. The start and end point of the CNVRs were analyzed for overlap with the start and endpoint of the genes with a minimum of 1 bp overlap. A 1 bp overlap was chosen due to the rough estimates of CNVR breakpoints and consequently some of the resulting overlapping genes located in the vicinity of the breakpoints could in fact be non-overlapping but in close proximity or alternatively be completely within the CNVR.

Analysis of the 72 CNVRs revealed that 38 CNVRs overlapped 148 genes (Additional file 1: Table S6) while the remaining 34 CNVRs contained no protein coding genes. Duplications overlapping genes outnumber deletions (8 deletions *vs*. 21 duplications) (Table 1), which is a similar observation as previously reported [31].

### GO analysis

The 148 1:1 orthologous genes affected by CNVRs (Additional file 1: Table S6) were analyzed with respect to gene enrichment using the g:GOSt tool from g:Profiler [41,42]. In the analysis, the human gene IDs corresponding to the canine genes were analyzed against all 16,305 1:1 human-dog orthologous genes that were used as background. The results were corrected for multiple testing using g:GOSt native method g:SCS. The most significant results in the domain molecular function was "olfactory receptor activity" (p = 1.0 × 10^-25^) and the Kyoto Encyclopedia of Genes and Genomes (KEGG) pathway enriched genes belonged to the "Olfactory transduction" pathway (p = 1.1 × 10^-23^) (Additional file 1: Table S7). This enrichment of olfactory receptor activity genes in CNVRs in dogs has been previously reported [31]. When all olfactory receptor genes were removed (totally 34/148 genes), no significant enriched category was obtained and in the domain molecular function the highest enriched gene category was "riboflavin transporter activity" with p = 2.34 × 10^-1^.

Of the stringent list of 15 single breed CNVRs, 7 CNVRs overlapped in total 20 genes of which 18 genes were in duplications and two genes were in deletions (Table 3). A gene enrichment analysis using the stringent list of 15 single breed CNVRs showed no statistically significant result neither when all 20 genes were analyzed nor when the 18 genes present in single breed duplications were analyzed separately. Of the two deleted genes, *PDE3B* was within the estimated CNVR boundary, and 14 of the 18 duplicated genes were within the estimated CNVR boundaries (Table 3, Additional file 1: Table S6). The CNVR, no. 78, encompassing *PDE3B* was found to affect 4/12 Finnish Spitz and in all individuals a heterozygous deletion was detected. This finding could be of importance for the occurrence of diabetes mellitus in this breed since knockout of the gene in mice has been shown to affect energy homeostasis, including altered insulin secretion and regulation of lipogenesis and lipolysis [43]. One interesting gene residing completely within the duplication CNVR no. 48, is *NOV* which is heterozygously duplicated along with additional ten genes in two dogs of the Standard Poodle breed (Table 3). *NOV* has been shown to have a selective pro-apoptotic activity towards adrenocortical cells in human and reduced expression and protein levels has been observed in childhood adrenocortical tumours [44]. These findings could have implications for Standard Poodles since this breed has a genetic predisposition towards Addison’s disease, which is characterized by insufficient production of hormones by the adrenal glands.

### Dosage sensitive genes and novel CNVRs

In a recent study of CNVRs in human, a number of human genomic regions were defined that hitherto do not contain any CNVRs despite numerously screened individuals [45]. These regions were denoted copy-number stable and contained 2312 human genes that were suggested to be dosage sensitive. The list of 148 canine genes (Additional file 1: Table S6) that were copy-number variable in the present study was investigated against the list of 2312 human dosage-sensitive genes in order to find possible important candidate genes for disease/phenotypic characteristics in dogs. This revealed that 23/148 canine copy-number-variable genes are suggested to be dosage sensitive in human (Table 4). Eleven of these genes are olfactory receptor genes and these are all located within one CNVR, no. 52, which is the CNVR that affect the highest number of breeds, namely 25/30 breeds. The remaining 12 genes were located in eight CNVRs that were all affecting not more than three breeds. Several genes could be of interest for future studies, *e.g*. one noteworthy gene is *SGPP2*, located in a duplication found in 10/11 Chinese Shar-Pei and in 4/11 Eurasian of all the screened breeds and was one of the rare CNVR that almost reached fixation in one breed. *SGPP2* is involved in inflammation and increased expression of the gene has been observed in skin lesions of patients with psoriasis suggesting that *SGPP2* may play a role in pro-inflammatory signaling [46]. This connection to inflammation could be of importance since the Chinese Shar-Pei breed is known to suffer from auto-inflammatory disease [29].

**Table 4:** Human-defined dosage sensitive genes within presently identified canine CNVRs

**CNVR nr**	**Chr**	**Start**	**Stop**	**Type**	Gene*	**Breed**	**No of affected individuals/total individuals**
2	1	65,361,010	65,528,809	del	TRDN	Elkhound	2/12
16	3	93,757,480	94,035,855	dupl	**ANAPC13**	English Bulldog	2/12
					LCORL		
21	4	74,490,811	74,617,835	dupl	**NUP155**	Golden Retr.Labrador Retr., Flat-coated Retr.	1/12 GR, 1/14 LR, 2/2 FR
					WDR70		
25	4	87,386,509	87,831,380	dupl	CDH18	Dachshund	4/12
41	11	12,650,626	13,811,903	dupl	**PRR16**	Bernese Mountain dog	2/11
52	14	5,305,030	5,752,740	del/dupl	**OR14C36**	25 breeds	totally 87 affected
					**OR14I1**	25 breeds	
					**OR2T1**	25 breeds	
					**OR2T11**	25 breeds	
					**OR2T2**	25 breeds	
					**OR2T27**	25 breeds	
					**OR2T29**	25 breeds	
					**OR2T35**	25 breeds	
					**OR2T4**	25 breeds	
					**OR2T5**	25 breeds	
					**OR2T6**	25 breeds	
69	19	13,816,712	15,228,649	dupl	PHF17	Chinese Shar-Pei	2/11
106	36	24,468,560	24,970,441	dupl	OSBPL6	Brittany Spaniels, English Setter	2/12 BS, 7/12 ES
					PDE11A		
					**RBM45**		
108	37	31,546,053	31,684,318	dupl	**SGPP2**	Chinese Shar-Pei, Eurasian	10/11 SP, 4/11 E

*Genes in bold are completely within the estimated CNVR boundaries, see also Additional file 1: Table S6.

In addition to the list of canine genes that have previously been seen to be dosage-sensitive in human [45], the present study identified 60 novel CNVRs where 31 were singletons and were therefore not considered in detail. The remaining 29 novel CNVRs (Additional file 1: Table S8) were not found in more than three breeds and 14 of these novel CNVRs were single breed CNVRs. There were 44 genes present in 15 of the 29 novel CNVRs and seven of these genes were suggested to be dosage sensitive in human, *e.g. SGPP2.* The list of novel CNVRs constitutes a resource for future studies.

## Conclusions

The present investigation is the first screen for canine CNVs using the CanineHD array and thereby this study demonstrates the performance of the CanineHD array in CNV detection in addition to SNP genotyping. The procedure described herein identified 72 CNVRs with high stringency where the smallest CNVR was 38 kb. A total of 29 novel CNVRs were identified that contribute to the catalogue of known canine CNVs and this also illustrates the importance of continuing the identification of canine CNVRs and the necessity of including a large number of samples and previously unscreened breeds in order to obtain a comprehensive catalog of canine CNVRs. Furthermore, a list of 15 confidently defined single breed CNVRs were found of which as many as 14 were novel CNVRs. These single breed CNVRs could be of interest for further genotype-phenotype analysis although the number of fixed single breed CNVRs are rare as previously shown [31]. Hence, breed-specific characteristics are most likely not hidden among hitherto unknown CNVs or alternatively are smaller than the CNV detection limit of approximately 50 kb on the CanineHD array. Nevertheless, there is still the possibility of having disease-predisposing CNVs among the growing list of defined CNVRs, which of course are much less likely to be fixed in a breed. A final contribution herein to future studies were the identification of 23 copy number variable canine genes that have been suggested as being dosage-sensitive in human and as such are remarkably interesting for future phenotype association analysis in dogs.

## Methods

### Samples and genotyping

A total of 359 dogs from 30 breeds were included in the study (Additional file 1: Table S1). This dataset was a subset of the "Gentrain" dataset that was used to initially evaluate the performance of the CanineHD 170 K SNP array with respect to SNP genotyping [35,36]. Blood sampling was performed by trained veterinarians according to relevant international guidelines and to the approval of the Swedish Animal Ethical Committee (no. C139/9 and no. C2/12). Genomic DNA was extracted and genotyped using the array according to manufacturer’s instructions. The initial step of the analysis, normalization of the total signal intensity and calculations of Log R ratio and B allele frequencies for the SNPs were performed in the GenomeStudio V2010.3 software package according to the manual and Peiffer *et al*. [47]. Furthermore, the Log R ratios were GC-corrected, adjusted for GC content surrounding the SNP as recommended to reduce waviness [48]. Subsequently, SNPs with a quality score of zero were excluded, likewise the intensity-only SNPs were removed since the analysis that followed could not manage these SNPs on other arrays than predefined human SNP arrays. This filtering left a total number of 172,115 SNPs for the CNV analysis (available through GEO Series accession number GSE55134) and at [49]).

### CNV discovery and quality filtering

The CNV discovery was performed using two programs; QuantiSNP and PennCNV. Both have been developed for CNV analysis on SNP array data and hence use both the Log R ratio and B allele frequency for each SNP in the CNV calling [23,24]. In QuantiSNP, default settings were used, *i.e.* L = 2 M, expectation-maximization iterations = 10, and the parameter file levels-hd.dat as recommended for high-density SNP arrays [23]. In PennCNV, a file containing the population frequency of B alleles was initially created using the samples within the present study. Thereafter the script "detect_cnv.pl" was used with the Hidden Markov Model file hhall.hmm [24].

The discovered CNVs were filtered based on quality information from PennCNV [24]. As recommended, a standard deviation of the Log R ratio below 0.25 and a waviness factor of >0.04 or < -0.04 were used to filter out samples with bad quality [38]. In addition, samples with 40 CNVs or more were also removed. This quality criteria was based on the observation that the number of CNVs per sample in the PennCNV file were ≤ 30 in 98% of the samples. Totally 8/359 samples did not fulfill the quality criteria and were removed from both the PennCNV and the QuantiSNP resulting CNV files. Subsequently, additional filtering was performed on the results file from both QuantiSNP and PennCNV, *i.e*. CNVs having less than 5 SNPs/CNV were excluded, as were CNVs on chromosome X. Also, three chromosomal regions were removed based on visual inspection of plotted Log R ratio due to extensive fragmented and noisy CNVs; chr1:13–15.7 Mb, chr15:10.2-13.5 Mb, chr36:16–19.2 Mb (Additional file 2: Figure S1, Additional file 1: Table S2). The QuantiSNP result file was finally filtered using a score, Log Bayes factor (LBF), calculated by the program that represents the support for the existence of the CNV. Two thresholds were used; CNVs with LBF < 10 were removed in the result file as recommended to give a maximum of 10% false positive calls, a threshold with LBF ≥ 10 was used to obtain a relaxed list of CNVs and a threshold with LBF ≥ 30 was used to obtain a stringent list of CNVs [23,37].

Subsequently, individual CNVs were merged into overlapping CNV regions (CNVR). Merging was performed separately on the result files from PennCNV and from the stringent list from QuantiSNP containing CNVs with LBF ≥ 30. The tool "Operate on genomic intervals" on the Galaxy platform was used for merging [50]. A CNVR was defined as in Redon *et al*., a region in the genome that can vary in copy number among individuals and the breakpoints of the CNVRs are the outermost boundaries of all the individual CNVs constituting each CNVR [4]. Thereafter, the QuantiSNP result file containing individual CNVs with a relaxed threshold of LBF ≥ 10 was used to genotype each sample for CNVs residing within the defined CNVRs. Hence, a single individual can have a different size of the CNV than the CNVR but it is located within the CNVR boundaries. The CNVRs were defined against CanFam 2.0.

### Comparison with published CNVRs and validation of CNVRs

To compare the performance of the CanineHD array in CNV detection and the described procedure to detect CNVRs, the results from two previously published CNV screens were used. The list of the herein identified CNVRs were analyzed for overlapping CNVRs from the two published datasets, using the tool "Operate on genomic intervals" on the Galaxy platform [50]. The two published datasets contain 394 CNVRs [31] and 1235 CNVRs [33] after removal of CNVRs on chromosome X and unknown.

Available DNA from 28 samples from three breeds allowed validation of nine CNVRs by quantitative real-time PCR (qRT-PCR) (Additional file 1: Table S4). The CNVRs were tested in breed/breeds where at least one individual per breed displayed a CNV, yielding a total of 106 sample-locus combinations (Additional file 1: Table S4). For each CNVR, one primer pair was designed in the centre of the CNVR based on the estimated boundaries obtained from the CanineHD CNVR discovery (primer sequences, see Additional file 1: Table S4). The qRT-PCR was performed in quadruplicates in a 10 ul volume using SYBR Green PCR Master Mix (Applied Biosystems) in a 7900HT Real Time PCR system (Applied Biosystems) according to manufacturer’s instructions. Final primer concentrations were 800nM and each primer pair was initially evaluated for amplification efficiency using a 5-point serial dilution curve with a 5X dilution factor. A dissociation curve analysis was performed after each run to assess PCR specificity. The 2^-∆∆Ct^ method was used for relative quantification of the copy number where the copy number assignment was obtained by the formula 2 x 2^-∆∆Ct^[51]. The ∆Ct is calculated for each individual by the average Ct (threshold cycle) of the tested CNVR – average Ct of a reference gene. The ∆∆Ct is given by the ∆Ct_tested individual_ - ∆Ct_reference individual_. The previously used *C7orf28B* was used as a reference gene (F: 5′-3′ CAACACAGGTTGACCAAGGA and R: 5′-3′ TTGTGCAGGATCAGAGCATC) [29]. The reference individual was chosen among the 351 samples and it displayed neither deletions nor duplications in the CanineHD CNV genotyping in any of the nine investigated CNVRs,

### Availability of supporting data

The data set discussed in this article has been deposited in NCBI’s Gene Expression Omnibus [52] and is available through GEO Series accession number GSE55134. The data set is also available at [49].

## Abbreviations

CNV: Copy number variant; CNVR: Copy number variant region; aCGH: Array comparative genomic hybridization; GWAS: Genome-wide association study; SNP: Single nucleotide polymorphism; LBF: Log Bayes factor; GC: Guanine cytosine; qRT-PCR: Quantitative real-time PCR.

## Competing interests

The authors declare that they have no competing interests.

## Authors’ contributions

AMM designed the study with advice from MTW and KLT. AMM performed the bioinformatic analysis and the validation experiment. JB contributed with bioinformatic support. AMM wrote the paper with support from JB, MTW and KLT. All authors read and approved the final version of the manuscript.

## Supplementary Material

Additional file 1: Table S1 Breeds included in the study. **Table S2.** Genomic regions and genes therein excluded from further CNV analysis due to noisy rawdata. **Table S3.** Summary of the identified 110 CNVR including the number of affected individuals per breed and the genotype for each of the 351 samples. **Table S4.** CNVRs selected for qRT-PCR validation, primer sequences and results from the qRT-PCR and the CanineHD genotyping. **Table S5.** The total list of 26 single breed CNVRs. **Table S6.** All 202 genes present in the identified 110 CNVRs, where the 20 genes present in the 15 strictly defined singlebreed CNVRs are in grey and the excluded 54 singleton genes are at the bottom of the table. **Table S7.** Results from GO analysis displaying significant p-values, *i.e.* 0.05 after multiple correction using g:SCS method. **Table S8.** Novel CNVRs that are present in >1 sample. Click here for file

Additional file 2: Figure S1 Illustration of the three genomic regions excluded from further CNV analysis due to heavy segmentation and noisy rawdata. Click here for file

Additional file 3: Figure S2 CanineHD Log R ratio and B allele frequency plots of two individuals with CNVR no. 45, which was not confirmed by qRT-PCR. Click here for file
