# Supplementary material for: Genome-wide copy number variant discovery in dogs using the CanineHD genotyping array
Source: BMC Genomics. 2014 Mar 19;15:210. doi: 10.1186/1471-2164-15-210 (PMC4234435; doi:10.1186/1471-2164-15-210)
Supplement: Additional file 2: Figure S1 — Illustration of the three genomic regions excluded from further CNV analysis due to heavy segmentation and noisy rawdata. [file 1471-2164-15-210-S2.pdf]

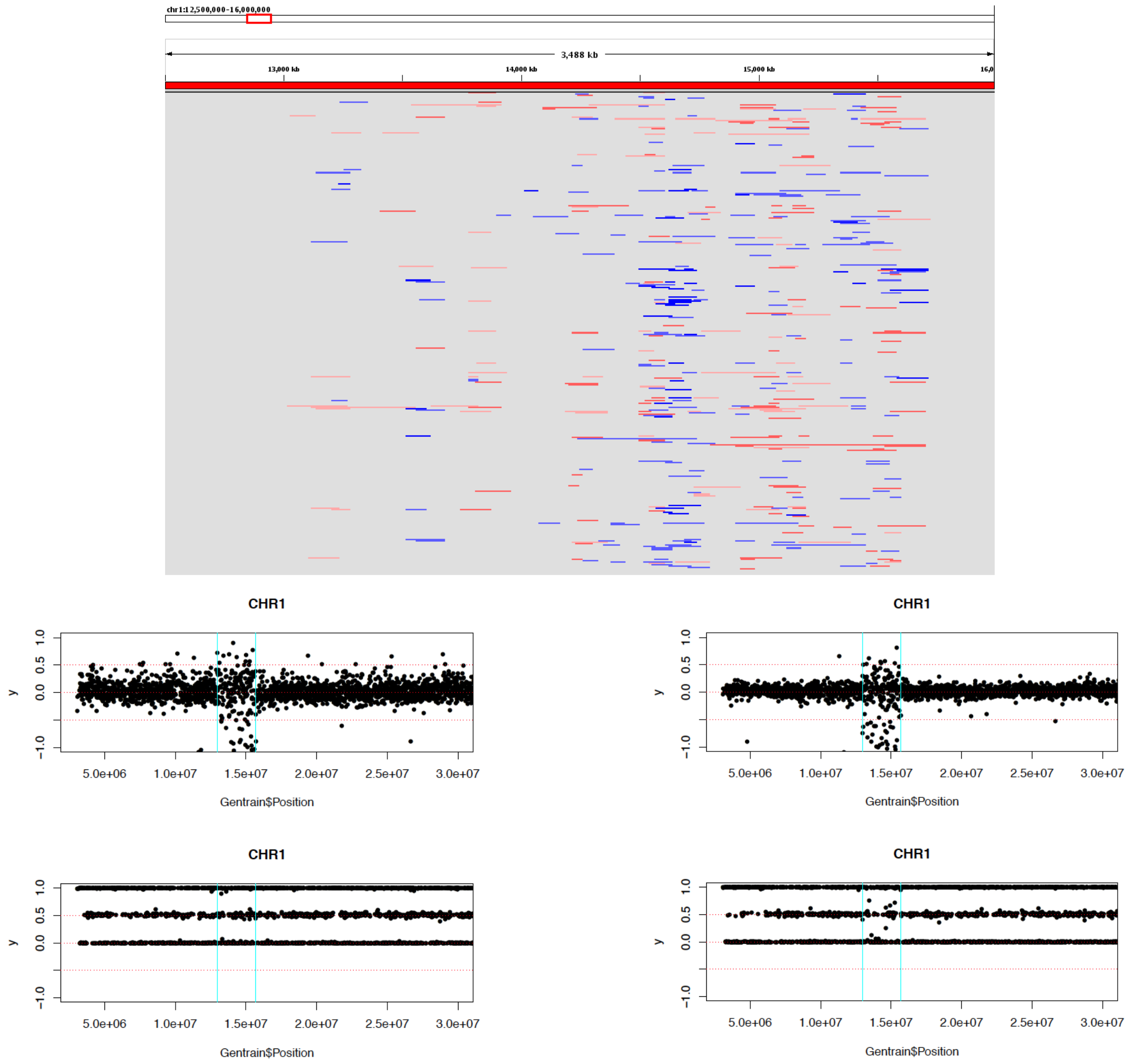

Figure S1A. The top panel shows a plot over the genomic region chr1:12,500,000-16,000,000 where the called CNVs in all 351 samples are illustrated as red bars for duplications and blue bars for deletions. The two bottom panels show the Log R ratio data (top) and the B allele frequency data (bottom) from two representative individuals. The light blue vertical lines in the bottom panels depict the region that was excluded from further analysis, i.e. 13,000,000-15,700,000.

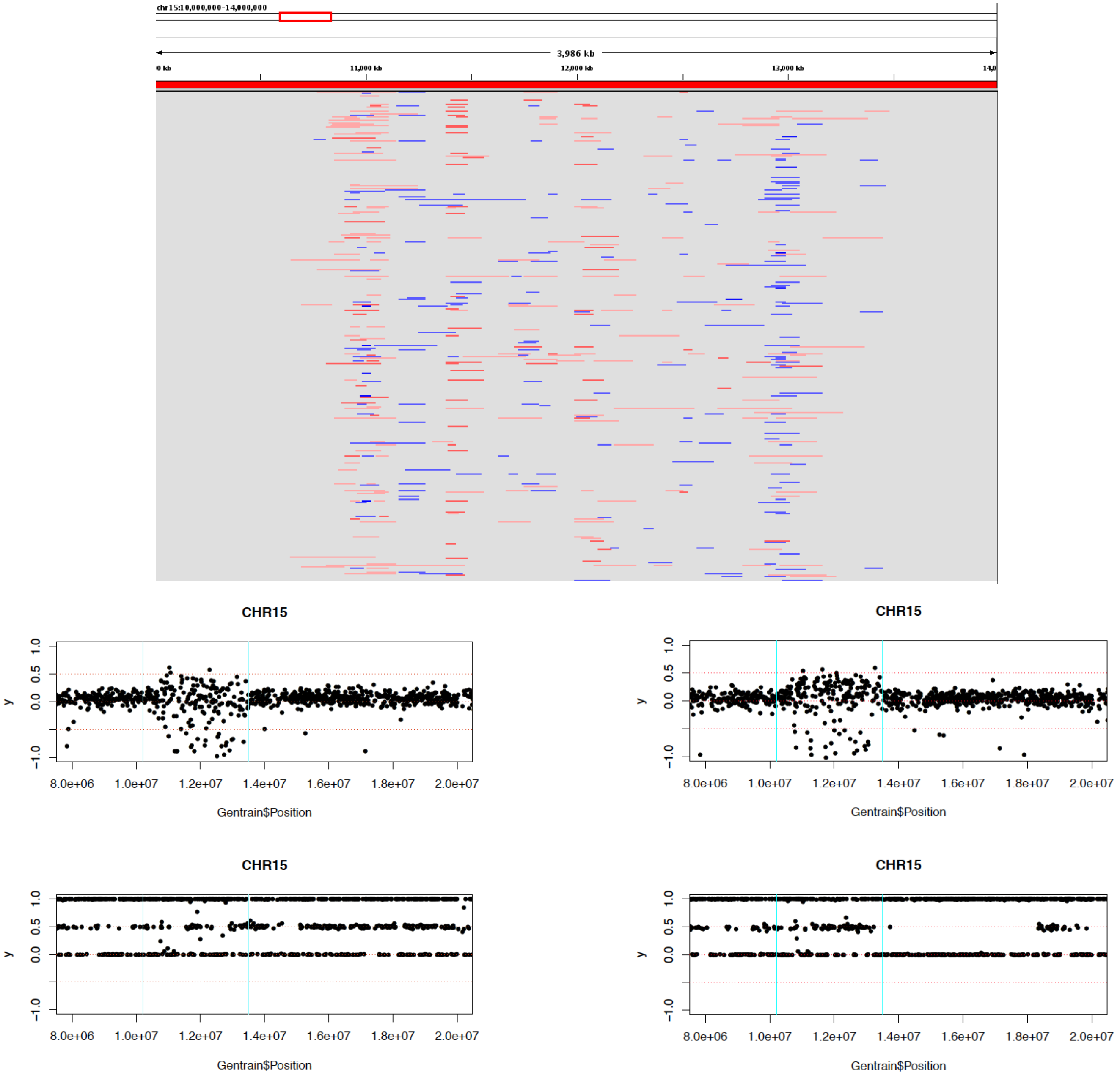

Figure S1B. The top panel shows a plot over the genomic region chr15:10,000,000-14,000,000 where the called CNVs in all 351 samples are illustrated as red bars for duplications and blue bars for deletions. The two bottom panels show the Log R ratio data (top) and the B allele frequency data (bottom) from two representative individuals. The light blue vertical lines in the bottom panels depict the region that was excluded from further analysis, i.e. 10,200,000-13,500,000.

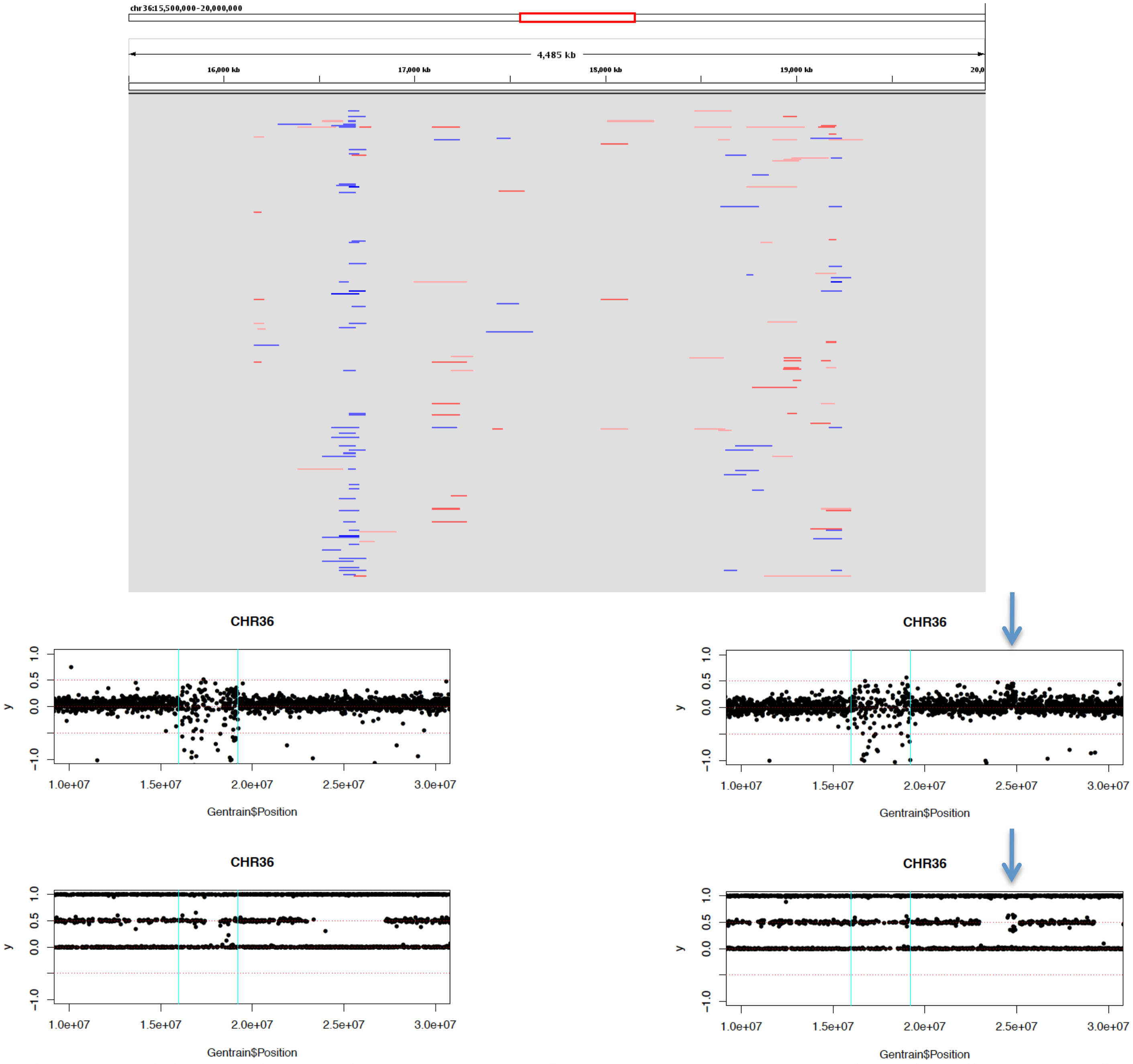

Figure S1C. The top panel shows a plot over the genomic region chr36:15,500,000-20,000,000 where the called CNVs in all 351 samples are illustrated as red bars for duplications and blue bars for deletions. The two bottom panels show the Log R ratio data (top) and the B allele frequency data (bottom) from two representative individuals. The light blue vertical lines in the bottom panels depict the region that was excluded from further analysis, i.e. 16,000,000-19,200,000. Arrows depict CNVR no. 106 that was one of the stringently identified CNVs.
