# Supplementary material for: Genome-wide copy number variant discovery in dogs using the CanineHD genotyping array
Source: BMC Genomics. 2014 Mar 19;15:210. doi: 10.1186/1471-2164-15-210 (PMC4234435; doi:10.1186/1471-2164-15-210)
Supplement: Additional file 3: Figure S2 — CanineHD Log R ratio and B allele frequency plots of two individuals with CNVR no. 45, which was not confirmed by qRT-PCR. [file 1471-2164-15-210-S3.pdf]

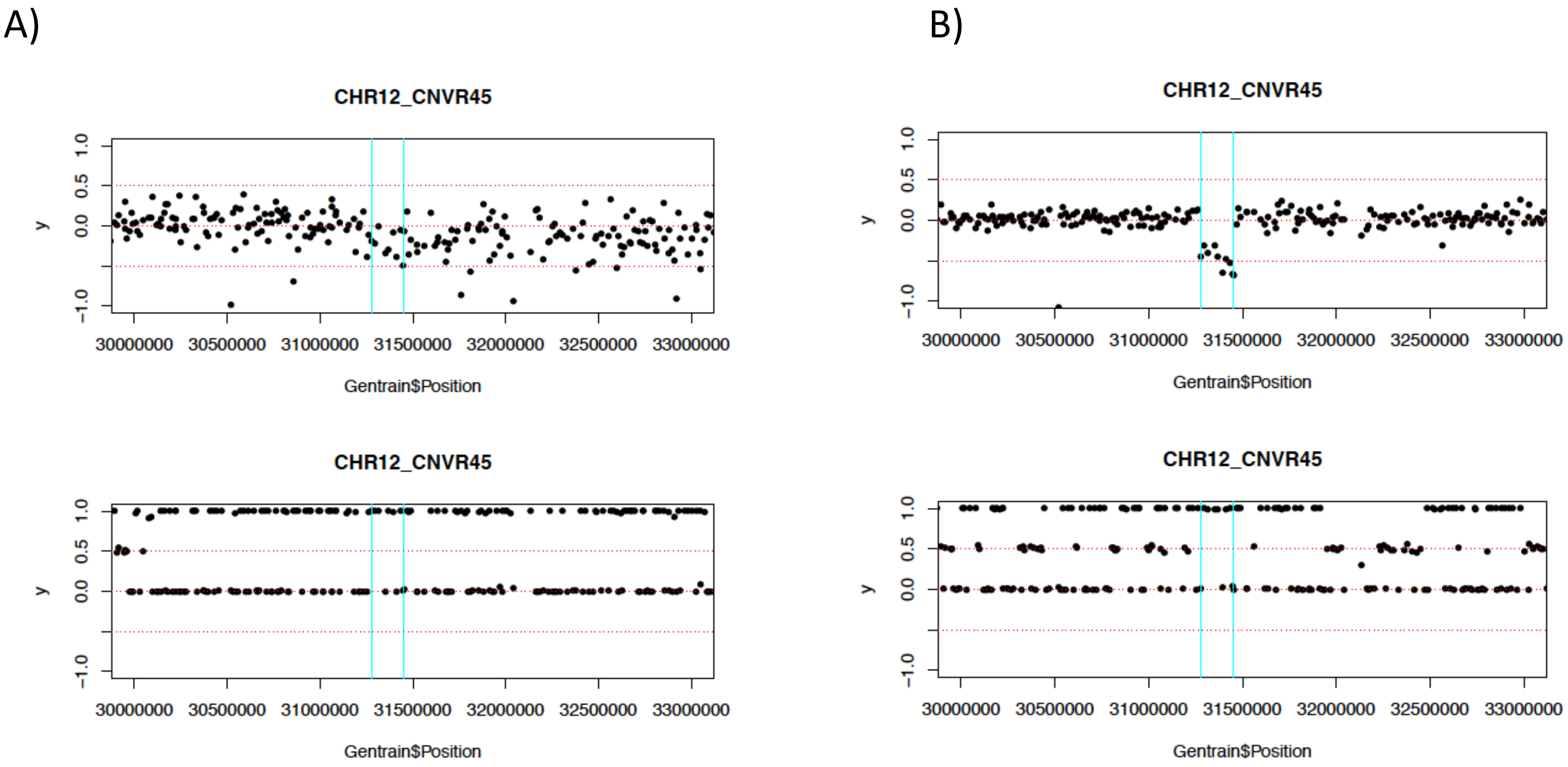

Figure S2. The two individuals genotyped as having CNVR no. 45 (a deletion). The two top panels show the Log R ratio data and the bottom panels display the B allele frequency data. The light blue vertical lines depict the boundaries of the CNVR. In a) the individual with the CanineHD genotyped deletion that however showed a normal copy number by qRT-PCR. In b) the second sample with the CanineHD genotyped deletion that was not available for qRT-PCR validation.
